# Engineering Targeted Gene Delivery Systems for Primary Hereditary Skeletal Myopathies: Current Strategies and Future Perspectives

**DOI:** 10.3390/biomedicines13081994

**Published:** 2025-08-16

**Authors:** Jiahao Wu, Yimin Hua, Yanjiang Zheng, Xu Liu, Yifei Li

**Affiliations:** Key Laboratory of Birth Defects and Related Diseases of Women and Children of MOE, NHC Key Laboratory of Chronobiology, Department of Pediatrics, West China Second University Hospital, Sichuan University, Chengdu 610041, China; wu_jiahao@stu.scu.edu.cn (J.W.); dryiminhua@scu.edu.cn (Y.H.); zhengyj@scu.edu.cn (Y.Z.)

**Keywords:** skeletal muscle disease, gene therapy, skeletal muscle target, AAV vector engineering

## Abstract

Skeletal muscle, constituting ~40% of body mass, serves as a primary effector for movement and a key metabolic regulator through myokine secretion. Hereditary myopathies, including dystrophinopathies (DMD/BMD), limb–girdle muscular dystrophies (LGMD), and metabolic disorders like Pompe disease, arise from pathogenic mutations in structural, metabolic, or ion channel genes, leading to progressive weakness and multi-organ dysfunction. Gene therapy has emerged as a transformative strategy, leveraging viral and non-viral vectors to deliver therapeutic nucleic acids. Adeno-associated virus (AAV) vectors dominate clinical applications due to their efficient transduction of post-mitotic myofibers and sustained transgene expression. Innovations in AAV engineering, such as capsid modification (chemical conjugation, rational design, directed evolution), self-complementary genomes, and tissue-specific promoters (e.g., MHCK7), enhance muscle tropism while mitigating immunogenicity and off-target effects. Non-viral vectors (liposomes, polymers, exosomes) offer advantages in cargo capacity (delivering full-length dystrophin), biocompatibility, and scalable production but face challenges in transduction efficiency and endosomal escape. Clinically, AAV-based therapies (e.g., Elevidys^®^ for DMD, Zolgensma^®^ for SMA) demonstrate functional improvements, though immune responses and hepatotoxicity remain concerns. Future directions focus on AI-driven vector design, hybrid systems (AAV–exosomes), and standardized manufacturing to achieve “single-dose, lifelong cure” paradigms for muscular disorders.

## 1. Introduction

Skeletal muscle, constituting the largest organ system and accounting for approximately 40% of total body weight in adults, functions not only as the principal effector of motor activities but also as a critical metabolic regulatory organ [1]. This specialized tissue exhibits both endocrine and paracrine capabilities, actively participating in systemic glucose homeostasis, lipid metabolism, and protein equilibrium through myokine secretion and substrate utilization [2,3,4,5,6]. Dysfunction of this tissue manifests in a spectrum of debilitating pathologies.

Hereditary myopathies, caused by pathogenic genetic variants, represent a significant category within skeletal muscle disorders. These conditions typically manifest as progressive muscle weakness involving skeletal and often cardiac tissue, frequently resulting in ambulatory impairment, respiratory compromise, and premature lethality [7]. These pathologies exhibit strong associations with specific genetic variants across multiple loci.

Dystrophinopathies, including Duchenne muscular dystrophy (DMD) and Beck muscular dystrophy (BMD), predominantly arise from variants in the X-chromosomal *DMD* (dystrophin) gene, leading to loss of dystrophin, which disrupts the critical dystrophin–glycoprotein complex (DGC) [8]. In contrast, Facioscapulohumeral muscular dystrophy (FSHD) is associated with epigenetic de-repression of the *DUX4* (double homeobox 4) gene, which leads to aberrant expression of the transcription factor DUX4 and cytotoxicity in skeletal muscle cells [9]. Limb–girdle muscular dystrophy (LGMD) exhibits significant genetic heterogeneity, being associated with variations in over 30 distinct genes. This diverse group is systematically classified into autosomal dominant (LGMD type D) and autosomal recessive (LGMD type R) forms based on the inheritance pattern of the pathogenic variants and the implicated genes. Further subcategorization defines specific subtypes according to the causative gene mutations. Notably, dominant subtypes include LGMD D2, caused by *TNPO3* (trasportin 3) mutations affecting TNPO3, and LGMD D4, arising from pathogenic variants in *CAPN3* (calpain 3) encoding CAPN3. Among recessive subtypes, key pathological entities comprise LGMDR2, resulting from *DYSF* (dysferlin) mutations leading to DYSF deficiency, and LGMD R3 through LGMD R6, which stem from mutations in the different sarcoglycan complex genes [10]. Myotonic dystrophy (DM) is the most common form of muscular dystrophy affecting adults. It consists of two types: DM1, associated with CTG repeat expansions in the 3′ untranslated region (3′ UTR) of the *DMPK* (dystrophia myotonica protein kinase) gene, and DM2, involving CCTG repeat expansions in intron 1 of the *CNBP* (cellular nucleic acid-binding protein) gene [11]. Congenital myopathies, presenting with hypotonia at birth or in infancy and motor developmental delay, include central core disease associated with *RYR1* (ryanodine receptor 1) gene variations, nemaline myopathy linked to *NEB* (nebulin) and *ACTA1* (actin alpha 1) gene variations, and other subtypes involving at least 13 additional genes [12].

In addition to variations in structural genes, hereditary myopathies are also caused by variations in genes involved in metabolism, mitochondrial function, ion channels, and distinct specific loci. Pompe disease linked to *GAA* (acid alpha-glucosidase) gene variations, Danon disease associated with *LAMP2* (lysosomal associated membrane protein 2) gene variations, and McArdle disease caused by *PYGM* (muscle glycogen phosphorylase) gene variations all exhibit defective glycogen catabolism [13,14,15]. Mitochondrial disorders arising from variations in mitochondrial DNA or nuclear DNA severely compromise cellular energy metabolism. Multi-system involvement manifests clinically in skeletal muscles as weakness, rhabdomyolysis, dysphagia, and related pathologies [16,17]. These conditions damage skeletal muscles through disrupted energy metabolism, pathological glycogen accumulation, and toxic metabolite generation. Ion channelopathies caused by mutations in voltage-gated ion channel genes, such as *CACNA1S* (calcium voltage-gated channel subunit alpha1 S), *SCN4A* (sodium voltage-gated channel alpha subunit 4), and *CLCN1* (chloride voltage-gated channel 1), disrupt calcium, sodium, and chloride homeostasis in skeletal myocytes. This impairs depolarization–repolarization cycles, leading to aberrant muscle excitability manifesting as periodic paralysis or myotonia [18]. Specific variants in *LMNA* (laminin) result in nuclear envelope disruption, DNA damage, and subsequent laminopathies, including Emery–Dreifuss muscular dystrophy (EDMD) and congenital muscular dystrophy [19].

Given this established genotype–phenotype correlation, gene-targeted therapies emerge as promising interventions to correct the underlying molecular pathology. Gene therapy encompasses the targeted delivery of therapeutic nucleic acids into specific cellular populations through sophisticated delivery modalities, facilitating precise genetic modification or functional protein restoration to address pathologies at their molecular foundation. As a promising therapeutic paradigm, 23 gene therapy products have secured global regulatory approval since 2000, addressing a diverse spectrum of conditions including monogenic disorders, hematologic malignancies, and select skeletal muscle pathologies [20].

Adeno-associated virus (AAV)-mediated systemic administration of microdystrophin, which functionally substitutes for dysfunctional dystrophin in DMD, demonstrates amelioration of muscle pathology, enhancement of contractile force, and attenuation of cardiomyopathy in preclinical models. Clinical investigations have demonstrated partial muscle preservation in DMD patients, evidenced by improved North Star Ambulatory Assessment (NSAA) scores, diminished creatine kinase levels, stabilization of muscular function, and decelerated disease progression [21,22]. Concurrently, antisense oligonucleotides (ASOs) modulate gene expression through selective binding of target mRNAs to rectify molecular aberrations [23]. In hereditary skeletal myopathies, ASO-mediated DNM2 suppression has demonstrated a reversal of myotubular myopathy in murine models, resolving histopathological abnormalities and restoring muscular strength [24].

Despite conventional gene therapy demonstrating therapeutic efficacy in hereditary muscular disorders, inherent limitations, including challenges pertaining to delivery efficiency, off-target effects, and immunological responses, continue to constrain its broader clinical translation and therapeutic potential. These unresolved impediments necessitate further innovation in vector architecture, target specificity, and immune modulation strategies to bridge the translational gap between experimental models and clinical implementation. Naturally occurring AAV serotypes employed in gene therapy exhibit non-selective tissue tropism [25]. High-dose AAV administration, associated with an elevated probability of serious adverse reactions, is frequently required to ensure optimal therapeutic outcomes. The systemic distribution of skeletal muscle presents substantial challenges for achieving comprehensive and uniform muscle targeting with conventional methodologies. For instance, effective DMD therapy necessitates systemic delivery to both skeletal and cardiac muscle tissues; however, current approaches demonstrate limited capacity to simultaneously target these anatomically distinct compartments [26]. The transgene capacity limitations of AAV vectors restrict large protein replacement strategies. Proteins deficient in essential functional domains exhibit suboptimal therapeutic efficacy compared to their native, fully structured counterparts. Furthermore, both transgene products and AAV capsids function as antigenic determinants in the human host, potentially eliciting immunological responses that limit delivery efficiency and therapeutic durability [27]. Recent years have witnessed concerted efforts toward enhancing targeting specificity in gene therapy applications.

In this review, we critically evaluate current strategies to augment targeting specificity in skeletal muscle gene therapy and summarize their respective advantages and limitations. Additionally, we address persistent challenges confronting contemporary gene therapy approaches and examine future developmental trajectories in this rapidly evolving field.

## 2. Skeletal Muscle: An Ideal Target for Gene Therapy

Skeletal muscle tissue exhibits inherent biological characteristics that contribute to its favored status as a target for achieving sustained transgene expression. Mature skeletal muscle fibers are functionally irreversible post-mitotic syncytia, having exited the cell cycle and thus largely forfeited mitotic capacity [28]. While postnatal muscle growth, maintenance, and repair primarily rely on satellite cells, the activation potential and contributions of this stem cell pool typically decline significantly with aging and muscle maturation [29]. Collectively, these properties result in relatively low cellular turnover within skeletal muscle tissue. Furthermore, the multinuclear architecture of myofibers confers a significant advantage for gene transfer; the successful transduction of even a single myonucleus within a fiber can enable robust expression of the transgenic protein. Importantly, expressed proteins often freely diffuse and distribute within the shared sarcoplasm of the syncytium. Consequently, proteins produced by modified nuclei can functionally compensate for deficiencies and provide support across the entire myofiber, potentially correcting metabolic or functional deficits in neighboring non-transduced regions [30]. Therefore, the convergence of myofiber post-mitotic stability, minimal intrinsic cellular renewal, and age-diminished satellite cell activity, along with the inherent ability of the syncytium to extend the functional benefit of transduced nuclei, synergistically supports the potential of skeletal muscle to sustain stable, long-term transgene expression, often reaching therapeutic levels. These features collectively render it a highly favorable target tissue for durable gene therapy approaches.

## 3. AAV Vector Development

**Figure 1:**
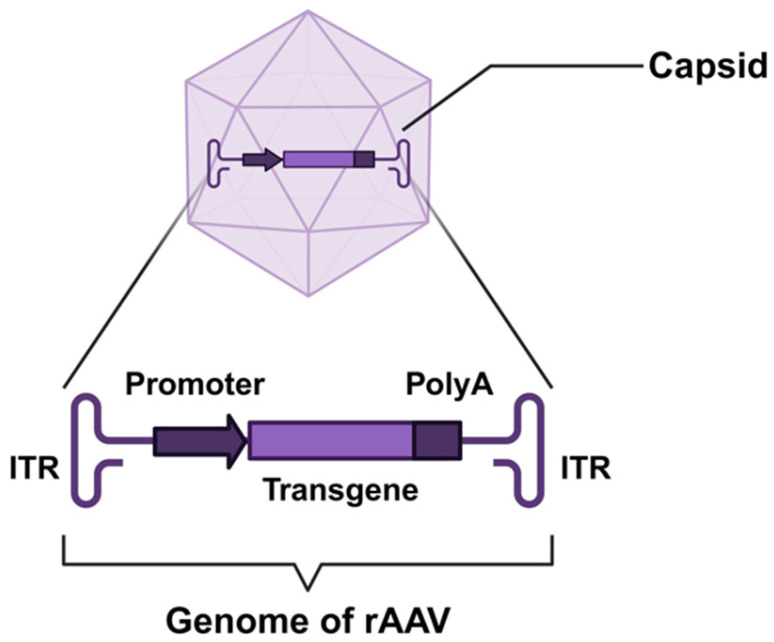
Schematic structure of recombinant adeno-associated virus (rAAV). The viral capsid encloses a single-stranded DNA genome flanked by inverted terminal repeats (ITRs). The key genetic cassette includes a promoter driving expression, the therapeutic transgene cassette, and a polyadenylation (polyA) signal sequence.

As the predominant gene therapy vector, AAV possesses an icosahedral protein capsid encapsulating a ~4.7 kb single-stranded DNA genome. This genome includes inverted terminal repeats (ITRs) crucial for replication and prolonged expression, while the capsid proteins (VP1/2/3) dictate tissue tropism through receptor binding [31]. Naturally occurring serotypes with skeletal muscle tropism include AAV1, AAV6, AAV8, and AAV9 [32]. However, their clinical utility for hereditary myopathies is limited by inadequate tissue specificity, off-target effects in non-muscle organs, inefficient transgene expression in skeletal muscle, dose-related immunogenicity, and pre-existing human immunity [33]. Based on current research, to overcome the limitations of naturally occurring AAV serotypes, AAV capsid engineering and genetic component optimization are employed to enhance their applicability in gene therapy for hereditary myopathies [34] (Figure 1).

### 3.1. Chemical Modification

Chemical modification of AAV capsid proteins entails the covalent coupling of chemical moieties, antibodies, or peptides to the surface of pre-existing AAV capsids, leveraging the inherent properties of conjugated molecules to modulate specific biological functions [35]. This approach fundamentally alters AAV tropism, immunogenicity, or detectability [36,37,38]. Contemporary AAV chemical modification strategies classify modifications based on targeted amino acid chemistry, distinguishing interventions directed toward canonical amino acids, such as lysine, cysteine, and so on, from those utilizing non-canonical amino acids (ncAAs) site-specifically incorporated via genetic code expansion technology. Intervention via ncAAs demonstrates superior site specificity compared to modifications targeting canonical amino acids, which face limitations in chemical reactivity and inherent lack of specificity. For instance, by strategically substituting surface residues in AAV-DJ capsids with an azide-bearing ncAA, biorthogonal click chemistry enables covalent conjugation of dibenzocyclooctyne (DBCO)-functionalized molecules to the capsid. Functionalizing AAV-DJ with folic acid–DBCO conjugates through this approach significantly elevated transduction efficiency for HeLa cells relative to unmodified controls [39].

Chemically modifying native AAV serotypes enables enhanced skeletal muscle tropism while concurrently reducing vector immunogenicity, presumably by neutralizing antibody evasion. Eric D. Horowitz et al. generated methylglyoxal-modified AAV2 (MGO-AAV2) via the glycation of surface-accessible arginine residues. This modification attenuated heparan sulfate’s binding affinity, redirecting viral tropism from hepatocytes to skeletal muscle and cardiac muscle in vivo [40]. María Stampa-López Pinto and colleagues covalently conjugated a modified poly (β-amino ester) (pBAE) polymer onto adeno-associated virus (AAV) capsids, yielding polymer-encapsulated vectors. Both in vitro and in vivo evaluations demonstrated significantly enhanced skeletal muscle tropism. Crucially, the polymer coating simultaneously prevented neutralizing antibody binding and reduced anti-AAV humoral immunogenicity [41]. These targeting and immunoevasive strategies advance AAV therapeutics for hereditary myopathies by concurrently improving delivery precision and addressing immunogenicity barriers.

Although chemical modification offers streamlined manufacturing and rapid production cycles, limitations include inherently non-specific reactions that risk off-target capsid modifications. Such unintended alterations may compromise infectivity, cause capsid misfolding, or yield heterogeneous vector populations [33,42]. Furthermore, residual exogenous moieties on the modified surface carry potential inflammatory or immunogenic risks [43].

### 3.2. AAV Capsid Engineering

**Figure 2:**
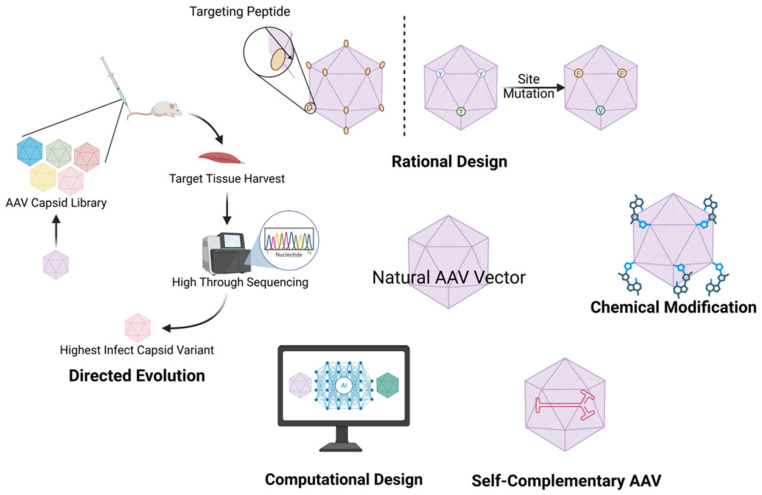
Engineering strategies for adeno-associated virus (AAV) vector optimization. This schematic summarizes five principal approaches to enhancing AAV vectors for gene therapy. Chemical modification: Covalent conjugation of functional moieties (e.g., polymers, peptides) to capsid surfaces, redirecting tropism and reducing immunogenicity. Rational design: Structure-guided genetic engineering of capsid proteins (e.g., peptide insertions, site-directed mutagenesis) to enhance tissue specificity and transduction efficiency. Directed evolution: Iterative selection of capsid variants from combinatorial libraries under physiological pressure to isolate mutants with improved muscle tropism and reduced off-targeting. AI-assisted engineering: Computational prediction of capsid–receptor interactions using machine learning (e.g., AlphaFold) and generative algorithms to design de novo capsids with tailored properties. Self-complementary AAV (scAAV): Genome engineering to enable double-stranded DNA packaging, bypassing second-strand synthesis and accelerating transgene expression kinetics.

Given that AAV serotype tropism is predominantly determined by the structural configuration of capsid proteins, bioengineering approaches to enhance vector specificity focus on rational modifications of these surface-exposed viral components. Contemporary AAV vector technology aims to transcend the inherent limitations of naturally occurring serotypes through sophisticated capsid protein engineering, thereby conferring enhanced tissue-specific tropism and diminished off-target transduction. The strategic modification of capsid’s architecture can be accomplished through three principal methodological approaches: directed evolution utilizing high-throughput screening platforms, rational design based on structural determinants of receptor binding, and combinatorial engineering employing chimeric or mosaic configurations of pre-existing serotypes (Figure 2).

#### 3.2.1. Rational Design

Chemical modification involves the direct covalent conjugation of molecules of interest onto pre-formed AAV capsid proteins, whereas rational design entails genetic engineering of the AAV *Cap* gene to enable the expression of such molecules as integrated fusion elements within the viral capsid [33]. The core of this technology lies in integrating the pathological microenvironment of target tissues and the characteristics of cell surface receptors to predict potential functional hotspots on the AAV capsid surface via three-dimensional (3D) structural modeling and molecular dynamics simulations, thereby guiding site-specific engineering for tailored modification.

Rational AAV design employs genetic engineering of the *Cap* gene to enable capsid display of tissue-targeting peptides, constituting a cornerstone strategy in vector retargeting. This paradigm leverages biomarker identification via transcriptome analysis, such as elevated *Itgav* and *Itgb6* expression in muscle tissue, pinpointing αVβ6 integrin as an optimal skeletal muscle transduction target. Capitalizing on this discovery, researchers engineered the chimeric serotype LICA1 through strategic insertion of a TGF-β-mimetic peptide into the VR-IV loop of a hybrid AAV9/AAVrh74 capsid. This modification exploits the natural high-affinity αvβ6–TGF-β interaction, simultaneously attenuating hepatic tropism while enhancing muscle-specific transduction. Functionally validated, low-dose LICA1 outperformed AAV9 by achieving sustained, supra-physiological microdystrophin overexpression in DMD mice and rescued the dystrophic phenotype in LGMD R3 models via α-sarcoglycan restoration, demonstrating significant therapeutic superiority [44].

Beyond peptide insertion, site-directed mutagenesis of solvent-exposed capsid residues offers a complementary engineering paradigm to reprogram tropism. Targeted tyrosine (Y) and threonine (T) substitutions, residues implicated in ubiquitin-mediated intracellular trafficking, demonstrably enhance AAV2 transduction efficiency [45]. Translating this mechanism to skeletal muscle targeting, researchers introduced analogous triple-point mutations (Y447F/Y733F/T494V; termed TM) into the AAVrh74 backbone, yielding substantially improved skeletal muscle transduction. Concurrent optimization of ITR regulatory elements revealed enhanced transgene expression mediated by the “S”-sequence within the D-region, enabling the development of high-expression genomes (GenX/GenY). The ultimate synergistic integration of engineered capsids (TM mutant) with optimized genomes (GenX/Y) yielded novel variants OptX and OptY, which demonstrated superior muscle tropism and transgene expression via combined evasion of proteasomal degradation and transcriptional potentiation [46].

Rational AAV vector design offers the compelling advantage of enabling precise tissue-specific tropism, reduced off-target transduction, enhanced transduction efficiency at lower doses, and improved therapeutic efficacy by strategically integrating targeting ligands or optimizing key capsid residues based on structural insights and pathophysiological cues, yet it suffers from the challenges of requiring complex multi-stage workflows (structural modeling, computational prediction, high-throughput screening), significant resource and time investments for discovery and validation, and inherent unpredictability regarding the impact of modifications on capsid stability, immunogenicity, and in vivo biological performance.

#### 3.2.2. Directed Evolution

While rationally designed AAV modifications fundamentally hinge on prior structural characterization of the capsid’s architecture and target tissues, empirical implementation frequently encounters critical data gaps. In such scenarios, achieving functional viral tropism requires systematic adoption of fallback strategies, including combinatorial library screening or directed evolution approaches [47]. This paradigm employs an iterative “mutation-screening-validation” cycle to overcome tissue-targeting bottlenecks. The methodology operates on two pillars: constructing comprehensive mutagenic libraries and conducting iterative biopanning under physiologically relevant selective pressures to progressively enrich variants with desired phenotypes. This framework surpasses natural diversity limits through triad library generation involving error-prone PCR for random point mutations, random peptide display for functional peptide insertions, and DNA shuffling for cross-serotype domain recombination. Subsequent multi-round screening under biologically relevant stressors drives phenotype enrichment, validated across species for clinical predictability.

In the Nagesh Pulicherla team’s evolution of AAV9, molecular structural analysis guided the selection of the AAV9.45 variant, which achieved balanced tissue specificity through maintaining cardiac/skeletal muscle transduction efficiency with concomitant hepatic off-target reduction [48]. Weinmann et al. leveraged peptide display and DNA shuffling library platforms for systemic in vivo screening in mice, with NGS-based organ biodistribution ranking yielding the functionally distinct AAVMyo variant, demonstrating approximately four-fold enhanced skeletal muscle transduction with minimal liver off-targeting [49]. Crucially, AAVMyo overcame natural AAV penetration limitations, achieving robust transduction in deep muscle groups (e.g., diaphragm, gastrocnemius) [49]. Beyond standalone directed evolution, combinatorial approaches integrate rational design with evolutionary strategies, exemplified by Tabebordbar et al.’s DELIVER platform. After in vivo screening yielded skeletal muscle-tropic MyoAAV1A—whose RGD motif leveraged integrin binding (strongest for αVβ6)—rational RGD insertion into its hypervariable loop VIII followed by DELIVER reselection generated optimized MyoAAV2A-2E variants [50]. Applied in cynomolgus macaques, this strategy produced primate-tropic MyoAAV3A-3E/4A-4E, with all novel serotypes validating therapeutic efficacy in murine DMD models. Similarly, El Andari et al. recombined muscle-tropic genomes into chimeric libraries, yielding primary H15/D20/S1/S10 variants; subsequent rational integration of first RGD and then P10 motifs (a tissue specificity enhancer) into these scaffolds generated AAVMYO derivatives with further optimized targeting—AAVMYO2/AAVMYO3 exhibited sharply reduced hepatic/cerebral off-target transduction and effectively alleviated both canine X-linked myotubular myopathy (XLMTM) via MTM1 overexpression and murine DMD via microdystrophin expression [51].

Notwithstanding its critical role in overcoming structural knowledge gaps, directed evolution offers both significant capabilities and inherent limitations for AAV vector engineering. Its major strengths lie in being a fundamentally knowledge-independent paradigm capable of accessing vast sequence diversity far beyond natural serotypes through combinatorial library construction (e.g., error-prone PCR, peptide display, DNA shuffling), crucially enabling the discovery of novel vectors with potentiated tissue tropism (e.g., AAVMyo penetrating deep muscle) and reduced off-targeting (e.g., liver escape in AAV9.45, MyoAAVs, AAVMYOs). The iterative in vivo biopanning process under physiologically relevant selective pressures allows for direct functional screening and stepwise enrichment of variants optimized for complex biological environments, demonstrating validated therapeutic efficacy across preclinical models. Furthermore, it synergizes effectively with rational design, as demonstrated by platforms like DELIVER, enabling both de novo discovery and subsequent iterative refinement (e.g., MyoAAV2A-2E/3A-4E, AAVMYO2/3). However, this approach demands substantial resources, high-throughput screening capacity, and extensive validation timelines due to its reliance on massive library generation and multi-round screening campaigns. The phenotypic success can be influenced by the specific selection pressures applied, potentially missing optimal variants if the screen conditions do not perfectly recapitulate the ultimate therapeutic context. Finally, while capable of generating potent candidates, the underlying molecular mechanisms driving the improved phenotype often remain empirical and require post hoc elucidation, limiting predictive design in future cycles. Nevertheless, as a powerful discovery engine for circumventing biological barriers and structural unknowns, directed evolution remains an indispensable tool for generating functionally superior AAV vectors with high translational potential.

#### 3.2.3. Computational Design

Contemporary AI-driven methodologies, such as the AlphaFold protein predictor tool, and machine learning (ML) strategies have catalyzed transformative advances in the mechanistic understanding of AAV capsid topology and vector–receptor interactomes while concurrently achieving order-of-magnitude enhancements in AAV engineering.

AI-empowered computational frameworks enable the prediction of AAV capsid binding thermodynamics, thereby establishing data-driven scaffolds for structure-guided vector engineering. Integrating AlphaFold-enabled structural proteomics with cryo-electron tomography (cryo-ET) interrogation establishes a macromolecular simulation platform, empowering AI-driven prediction of AAV tropism landscapes via organ-specific receptor expression profiling and enabling precision retargeting through allosteric pocket engineering. Through this AI-based platform, Timothy F. Shay et al. mechanistically delineated the allosteric binding interface between human IL-3/LPR6 receptors and AAV9 capsid proteins [52]. This structural elucidation provides atomic-level insights into complement activation-mediated immune responses in vivo and engineered capsid variants capable of potentiated blood–brain barrier penetrance. Ai Vu Hong et al. identified the integrin αVβ6 heterocomplex as a highly expressed receptor in cardiac/skeletal muscle and then rationally engineered an AAV capsid by substituting its VR-IV loop with an αVβ6-targeting motif. Computational validation confirmed high-affinity binding, yielding the muscle-tropic vector LICA1 [44]. Deep-learning-enabled reverse engineering of directed-evolution-derived AAV variants deciphers structural determinants for tissue-specific tropism. Atomic-level reconstruction of capsid architectures reveals epistatic interactions governing tropism, thereby establishing mechanistic blueprints for rational viral vector engineering. Structural landscapes of skeletal muscle-tropic AAV variants (LICA1, AAVMYO, MyoAAV) reveal a conserved structural alteration, the convergent insertion of RGD motifs within the VR-IV (residues 451-464) and VR-VIII (587-600) loop regions, which can be applied in future skeletal muscle targeting AAV engineering [44,50,51].

Beyond structural characterization, machine learning (ML) has fundamentally transformed AAV capsid engineering by overcoming the limitations of traditional directed evolution [53]. Conventional random mutagenesis for generating functional AAV variants exhibits low efficiency due to an inability to ensure the structural integrity of mutated variants and limitations in screening technologies. In contrast, ML algorithms, including supervised, unsupervised, and reinforcement learning, enhance AAV evolution efficiency by learning from existing AAV capsid protein sequences and their features to predict critical properties of library variants (e.g., tissue tropism, biodistribution, and immunoreactivity), thereby enabling targeted experimental validation of predicted high-potential candidates to obtain desirable variants [53,54,55]. Although ML-based techniques have not yet been applied to enhance skeletal muscle tropism, several groups have successfully improved CNS targeting using ML models (e.g., Transformers, neural networks) [56,57]. Notably, the Fit4Function platform, developed by Fatma-Elzahraa Eid et al., has achieved cross-species validation for enhanced liver transduction [58]. These advances underscore ML’s potential for developing diverse targeted delivery systems across multiple organs/species. The incorporation of deep learning enables the generation of AAV libraries with enhanced structural diversity, significantly expanding the repertoire of candidates for downstream AAV screening pipelines [57,59]. While ML has substantially accelerated AAV capsid evolution, critical challenges remain for future investigation. These include extending this technology to enhance tropism for additional organs and improving the sequencing fidelity for AAV capsids, particularly sensitivity for detecting low-abundance variants, to broaden ML’s utility in AAV engineering. Furthermore, the current reliance on specialized infrastructure and high technical resource demands limit the widespread adoption of ML-driven AAV development.

### 3.3. Self-Complementary AAV Vectors

Self-complementary AAV (scAAV) is a genomically engineered AAV variant. Unlike conventional single-stranded AAV (ssAAV), scAAV forms a covalently closed double-stranded DNA structure capable of immediate transcription initiation without requiring host-cell-mediated DNA synthesis [60]. In diverse target tissues, including skeletal muscle, scAAV demonstrates superior transduction efficiency and accelerated transgene expression kinetics relative to ssAAV, attributable to its pre-formed double-stranded DNA genome [61].

The enhanced transduction efficiency of scAAV has facilitated its utilization in developing gene therapies for a broad spectrum of hereditary myopathies. Yu Zhang et al. achieved exon 45 skipping in *dmd*^ΔEx44^ mice using scAAV-packaged sgRNA combined with ssAAV-Cas9, restoring dystrophin expression and muscle function, with the scAAV-sgRNA dose reduced by at least 20-fold compared to the ssAAV required for equivalent editing efficiency [62]. Beyond gene editing applications, scAAV demonstrates significant optimizations for gene replacement therapy by enabling rapid, high-level transgene expression. Prenatal administration of SMN-expressing scAAV9 prevented motor neuron loss and muscle atrophy in a spinal muscular atrophy (SMA) mouse model, demonstrating significantly enhanced transduction efficiency over ssAAV9 [63]. Systemic delivery of scAAVrh74-MHCK7.hSGCB achieved muscle-specific transgene expression in LGMDR4 mice, substantially ameliorating dystrophic pathology while demonstrating long-term therapeutic transgene persistence without vector-related toxicities [64]. Despite enabling robust transgene overexpression, scAAV’s genome ≤ 2.3 kb packaging constraint necessitates ultra-compact expression cassettes for larger therapeutic genes and precludes the delivery of oversized transgenes. However, scAAV’s double-stranded DNA genome presents a double-edged sword; while enabling rapid transgene expression, the dsDNA structure potently triggers innate immune responses [65]. Furthermore, scAAV’s complex manufacturing hurdles, including inefficient capsid assembly, currently preclude large-scale cGMP production. These limitations constitute critical barriers to clinical translation despite its therapeutic promise.

## 4. Genetic Cassette Engineering

**Figure 3:**
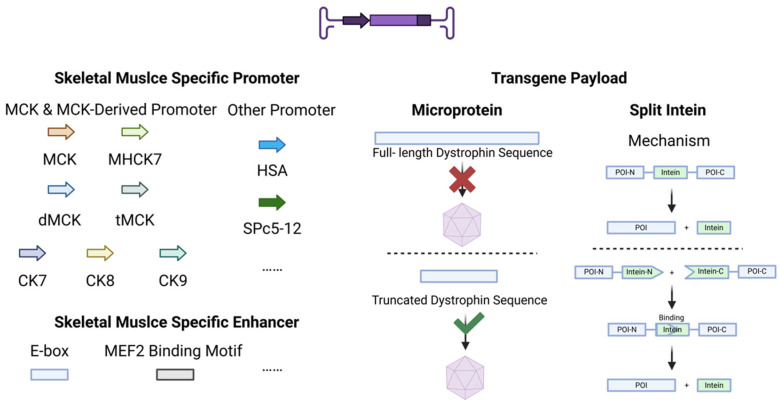
Engineering of AAV gene expression cassettes for muscle-specific therapy. This schematic illustrates key strategies to optimize AAV transgene cassettes for skeletal-muscle-targeted gene delivery. Muscle-specific promoters: MCK-derived promoters (e.g., full-length MCK, tMCK etc.) drive high-fidelity expression in striated muscle. Alternative promoters (e.g., *HSA* for human α-actin, *SPc5-12* synthetic promoter) enable tailored transcriptional activity and reduced off-targeting. Skeletal-muscle-specific enhancers: E-box motifs (CANNTG) bound by myogenic transcription factors (e.g., MyoD) enhance spatial specificity, and MEF2-binding sequences recruit regulators of muscle development, amplifying transgene expression while maintaining tissue restriction. Transgene payload engineering, microprotein variants: Design of functional miniaturized proteins (e.g., microdystrophin ΔR4-R23/ΔCT) to overcome AAV packaging limits (~4.7 kb). Split intein systems: Co-delivery of split-intein-fused protein fragments that undergo in vivo reconstitution into full-length functional proteins (e.g., POI: protein of interest).

Beyond the AAV capsid, the viral genome of recombinant AAV vectors constitutes another critical determinant of viral tropism. The recombinant genome typically encompasses regulatory elements, such as cis-regulatory elements, including promoters/enhancers/silencers/regulatory sequences, transgenic payloads, WPRE, and poly-A tails (Figure 3).

### 4.1. Promoters

Tissue-specific promoters represent a critical advancement in gene therapy, confining transgene expression to target cell populations and mitigating systemic immune responses. Skeletal-muscle-specific promoters exemplify this precision, enabling sustained sarcolemmal-localized expression while circumventing off-target transcriptional activation, thereby enhancing therapeutic safety profiles [66].

The murine muscle creatine kinase (MCK) promoter constitutes a foundational element in this domain. Its transcriptional specificity is mediated through E-box cis-regulatory elements bound by core myogenic factors (e.g., MyoD), driving expression exclusively in skeletal muscle tissue. This specificity facilitated therapeutic success in preclinical models, as AAV-MCK-driven miR-486 delivery ameliorated functional deterioration in MMTV-Neu transgenic mice, rescuing impaired isometric contraction strength and motor deficits [67]. Crucially, Rui Xu et al. demonstrated that MCK-directed GALGT2 expression unexpectedly mitigated dystrophic cardiomyopathy in mdx models, revealing intrinsic dual cardioskeletal tropism, a discovery with profound implications for Duchenne muscular dystrophy (DMD) management given its cardiac manifestations [68].

Notable refinements to the MCK architecture have emerged. The chimeric MHCK7 promoter—a fusion of MCK and α-myosin heavy chain enhancer elements—achieves superior specificity across myofiber subpopulations and cardiac tissue while reducing hepatic off-target expression, enabling its deployment in multiple clinical-stage microdystrophin trials [69,70]. Engineered truncations incorporating synthetic E-box elements (CK6, dMCK, tMCK) reconstitute transcriptional potency; for instance, rAAV9-tMCK-mediated DOK7 overexpression sustainably corrected neuromuscular junction pathology in skeletal muscle with minimal ectopic transduction [71,72]. Further optimization through spatial reorganization of regulatory motifs developed the CK-class promoters (e.g., CK7/CK8), enhancing cardioskeletal fidelity. Application of CK8 achieved multi-tissue therapeutic efficacy in mdx mice, durably preventing functional decline in respiratory and limb musculature [73,74].

Beyond MCK-derived systems, the human α-actin promoter (ACTA1) demonstrates robust skeletal-targeted activity, as validated by PFKFB3 overexpression improving post-ischemic neuromuscular recovery [75,76]. Similarly, the engineered SPc5-12 promoter derived from the combinatorial integration of muscle-specific enhancers with a chicken α-actin core enabled high-fidelity microdystrophin and α-sarcoglycan expression, providing therapeutic rescue in DMD and LGMDR3 disease models [66,77,78].

### 4.2. Enhancers

Enhancers potentiate the transcription of target genes through multifaceted mechanisms, including direct recruitment of lineage-specific transcription factors to form active transcriptional complexes and the facilitation of chromatin remodeling. The inherent sequence specificity of enhancers, combined with the cell-type-restricted expression of cognate transcription factors, mechanistically underlies their precise tissue-selective activity [79]. Skeletal-muscle-specific enhancers encompass classical cis-regulatory elements, such as E-box sequences (CANNTG), that serve as binding platforms for myogenic transcription factors, along with consensus MEF2-binding motifs (CAGCTG), which collectively mediate transcriptional specificity in myogenic lineages [80,81].

As delineated, skeletal-muscle-specific enhancers (e.g., E-box-containing elements) operationalize transcriptional enhancement through synergistic assembly with cognate promoters into cis-regulatory modules. This cooperative architecture potentiates the fidelity of gene expression restricted to the skeletal myocyte lineage. Current clinical imperatives to augment therapeutic transgene expression necessitate de novo identification of synergistic enhancer–promoter constellations. This represents a critical modality for enhancing targeting fidelity within the skeletal myocyte compartment. S. Sarcar et al. employed genome-wide in silico screening to identify multiple cis-regulatory modules harboring myogenic transcription factor binding motifs. By synergistically coupling these modules with canonical promoters (SPc5-12, Des), they engineered skeletal myocyte-optimized cis-regulatory architectures exhibiting approximate 400-fold enhanced transcriptional efficiency over conventional systems. Functional validation was achieved through therapeutic human microdystrophin and follistatin expression, which ameliorated dystrophic pathology in murine models [82]. This pioneering work establishes a modular expression cassette platform for skeletal-muscle-directed gene therapeutics.

### 4.3. Transgene Payload

Due to the restrictive threshold (~4.7 kb) imposed by the ITR sequence of AAV genomes for maximum packaging capacity, full-length proteins with high molecular weight, such as dystrophin, cannot be delivered via a single-AAV vector system. To overcome the packaging constraints of AAV for large therapeutic proteins, two strategies have emerged: rational design of multi-functional microprotein variants and split intein-mediated trans-splicing.

As an exemplar macromolecular therapeutic protein, dystrophin has prompted the development of multiple engineered truncations designed to accommodate AAV packaging constraints. Functional analysis of dystrophin’s amino acid sequence enables rational design of diverse truncated variants retaining critical domains that have already been applied in clinical use, though substantial therapeutic heterogeneity persists among different engineered constructs. Julian N. Ramos et al. engineered eight dystrophin truncations that achieved uniform skeletal muscle expression but exhibited differential efficacy in nNOS expression and force recovery [74]; concurrently, Cora C. Hart et al. demonstrated that four clinical-stage microdystrophin constructs rescued hereditary myopathy in *mdx* mice, while two variants paradoxically induced cardiomyopathy characterized by impaired cardiac contractility and exacerbated myocardial fibrosis [83]. This renders rigorous protein structural engineering coupled with experimental validation imperative for developing optimized truncated variants.

The discovery of inteins and the subsequent application of split intein technology have provided a breakthrough solution to this challenge. Inteins are naturally occurring protein splicing elements that catalyze protein splicing [84]. Following self-excision, the intein seamlessly joins the flanking extein sequences via a peptide bond. Split inteins natively exist as two separate subunits: the N-intein and C-intein fragments. Binding of these two halves triggers a splicing reaction that ligates the extein domains [85]. This approach enables the partitioning of large protein sequences into two segments, both independently within the 4.7 kb packaging limit of AAV vectors. These segments are fused with split intein sequences and packaged into separate AAV particles. Upon co-delivery in vivo, the reconstituted split intein mediates protein splicing, enabling the reconstitution of full-length functional proteins.

In 2024, Yuan Zhou et al. employed two artificially optimized split inteins with high-efficiency binding and splicing capabilities, combined with skeletal-muscle-tropic AAV serotypes and tissue-specific promoters. Through triple AAV vector delivery in mice, they achieved tissue-restricted expression of full-length dystrophin specifically in skeletal muscle and successfully rescued the phenotype of DMD mice [86,87]. The study further revealed that compared to microdystrophin therapy for DMD, full-length dystrophin effectively restores the sarcolemmal localization of cavin-4 while concomitantly mitigating aberrant activation of the associated ERK1/2 signaling pathway. The study establishes that the expression of full-length proteins containing all functional domains is essential for comprehensive disease phenotype remission, demonstrating marked therapeutic advantages over truncated protein approaches. Beyond delivering therapeutic target proteins, split intein technology thereby affords the possibility of delivering large-molecular-weight tool proteins essential for advanced genomic interventions. Split-intein technology enables skeletal-muscle-specific expression of high-molecular-weight tool proteins, including Cas9, base editors (ABE/CBE), and prime editors, via dual AAV delivery platforms. The successful application of gene editing tools has enabled exon skipping or direct correction of nonsense mutations in the dystrophin gene, ultimately restoring dystrophin expression in human, porcine, and murine DMD models [88,89,90].

## 5. Non-Viral Vectors

### 5.1. Liposomal Vectors 

Liposomes are phospholipid bilayer-based nanovesicles with a hydrophobic membrane and hydrophilic core, enabling the co-delivery of both hydrophilic and hydrophobic drugs [91,92]. Owing to the natural presence of phospholipids in cell membranes, liposomes exhibit superior biocompatibility and safety profiles [93]. Their surface can be functionalized with antibodies or chemical moieties to confer target specificity [94]. These attributes establish liposomes as promising delivery vectors for gene therapy.

Multiple engineered liposomes with skeletal-muscle-targeting capabilities are now actively developed in current research. Functionalization of liposomes with the α-DG-targeting peptide A2G80 (derived from laminin) enabled skeletal-muscle-selective delivery, as demonstrated by Sasaki’s team in DMD preclinical models [95]. By conjugating skeletal-muscle-specific receptors—including mannitol-modified LRP3, MyHC-2A, and MYF5; leucine-modified MYOG; and azide-functionalized MYHC-2B—to lipid nanoparticles, Yingqian Wang et al. enhanced carrier targeting to skeletal muscle. This approach effectively addressed abnormal lipid deposition via the overexpression of miRNA-130a [96]. Eriya Kenjo et al. developed novel skeletal-muscle-targeting liposomal carriers with low immunogenicity using combinatorially engineered lipids. These vectors enabled functional Cas9 protein expression in humanized DMD model mice, restoring dystrophin through exon skipping while demonstrating robust transduction efficiency across all major limb myofiber groups [97]. Targeted liposomal design significantly enhances skeletal muscle delivery precision through receptor-mediated active targeting mechanisms. Engineering modifications have expanded liposomal functionality, while progressive enhancement in targeting efficacy has been achieved with advanced material development. Combining diversified materials enables tailored customization with multiple desirable properties. Moreover, synergistic low immunogenicity and simplified manufacturing processes drive their emergence as clinically viable candidates.

### 5.2. Polymeric Vectors

Polymeric delivery vectors are nanoscale gene delivery systems engineered from chemically synthesized or naturally derived polymer materials. Through electrostatic complexation or covalent conjugation, these vectors encapsulate nucleic acids (DNA, mRNA, siRNA, CRISPR components) to achieve targeted delivery and controlled release [98].

Manuela Chiper et al. and Shani Attias Cohen et al. utilized a hydrogel-based polymeric carrier in the form of injectable PEG-fibrinogen (PF) microspheres for the delivery of chemically modified 2′-O-methyl phosphorothioate (2OMePs) antisense oligonucleotides (AONs), achieving exon-skipping-mediated restoration of dystrophin expression in the skeletal muscle of DMD model mice [99,100]. Michael R. Hicks, Xiangsheng Liu, et al. injected novel mesoporous silica nanoparticles covered by a phospholipid bilayer into wild-type and multiple DMD model mice. They found that these nanoparticles preferentially distributed to muscles exhibiting dystrophic phenotypes. This distribution pattern was independent of blood flow or immune status, demonstrating their dual potential for diagnosing DMD muscle atrophy and delivering targeted DMD therapeutics [101]. While polymeric materials exhibit high programmability, enabling multimodal customization for specific objectives, their biocompatibility varies significantly depending on constituent polymers. Suboptimal intracellular transfection efficiency remains an additional constraint, necessitating systematic optimization of these parameters for future applications.

### 5.3. Extracellular Vesicles/Exosomes

Exosomes, natural nanovesicles from diverse cells, offer significant gene therapy potential for hereditary myopathies. Their inherent biocompatibility, targeted delivery, and low immunogenicity overcome limitations of conventional systems with poor penetration and high immunogenicity [102].

They effectively protect and deliver therapeutic nucleic acids or proteins to repair mutations or restore protein function, ameliorating symptoms [102,103]. Exosomes achieve muscle tissue targeting via surface proteins (e.g., CD63), and CD63 modification could enhances muscle cell uptake and gene delivery efficiency [104]. Crucially, in DMD, myotube-derived exosomes in particular enhance sarcolemmal integrity and stabilize the dystrophin complex by inhibiting Ca^2+^ influx and calpain activation, mitigating muscle degeneration [105]. In DMD gene therapy, systemic delivery of exosomes loaded with splice-correcting oligonucleotides (EXO_EAA-PMO_) to *mdx* mice demonstrated superior efficacy over free PMO, enhancing dystrophin restoration in quadriceps by up to 18-fold and improving muscle function [106]. Moreover, allogeneic muscle progenitor cell-derived exosomes (MPC-EXO) concurrently ameliorated DMD cardiac dysfunction through anti-inflammatory and anti-apoptotic effects [107]. For currently untreatable dysferlinopathy, exosomes from myotubes or human serum act as potent nanocarriers, delivering full-length dysferlin gene/protein to deficient fibers and restoring dysferlin expression, offering a novel therapeutic avenue [103].

To overcome inherent loading limitations, engineering strategies were developed. CP05 modification enables high-efficiency PMO loading [104], while exosome–liposome hybrid systems increase cargo capacity while retaining targeting specificity [108]. Exosomes can be combined with AAV to create dual-vector delivery platforms, maintaining AAV’s high transduction efficiency while circumventing neutralizing antibodies [108]. The success of this technology in cardiac muscle holds promise for its application in skeletal muscle gene therapy [109]. And, fusion with CD63 or muscle-homing peptides further enhances the targeting precision of exosomes [104]. Although promising, optimizing in vivo targeting and dosing protocols remains crucial.

### 5.4. Physical Enhancement of Delivery

**Figure 4:**
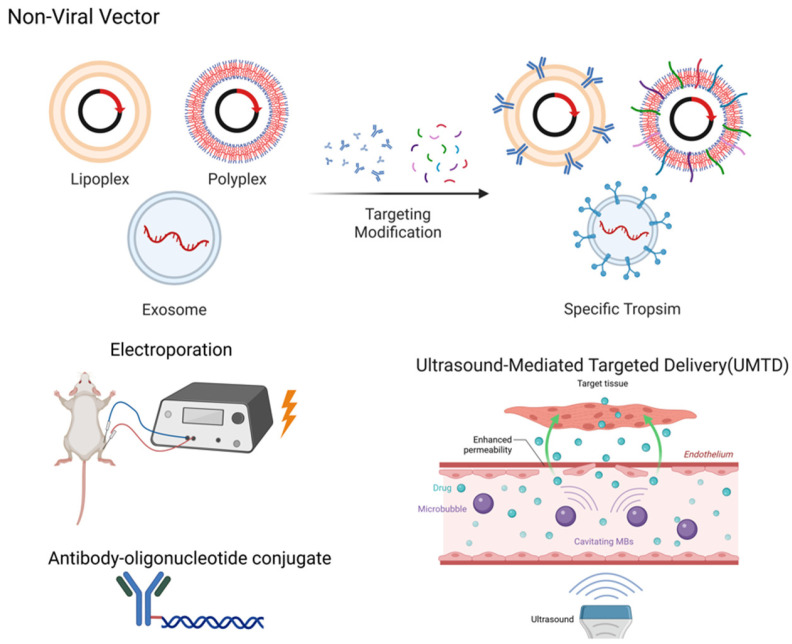
Non-viral delivery strategies for skeletal-muscle-targeted gene therapy. Functionalized nanocarriers, liposomes/polymeric nanoparticles/exosomes: Surface conjugated with muscle-targeting ligands (e.g., integrin αVβ6-binding peptides, myosin-specific antibodies) to enable receptor-mediated uptake. Physical enhancement methods, electroporation: Transient electrical pulses increase sarcolemmal permeability, boosting plasmid/nucleic acid uptake in myofibers. Ultrasound-mediated targeted delivery: Microbubble cavitation under focused ultrasound disrupts vascular endothelia, enhancing the extravasation and penetration of nanoparticles (e.g., LNP-ASOs) into deep muscle groups. Antibody–oligonucleotide conjugates (AOCs): Covalent linkage of antisense oligonucleotides (ASOs) or siRNAs to skeletal-muscle-specific antibodies enables antibody-directed endocytosis and precise intracellular delivery.

In skeletal muscle gene delivery, physical enhancement methodologies substantially increase the transfection efficiency of plasmids and naked DNA while synergistically augmenting transduction efficiency and therapeutic outcomes across diverse vector systems (Figure 4).

Localized electroporation transiently increases sarcolemmal permeability to facilitate exogenous gene delivery into skeletal myofibers. This approach elevates naked DNA expression in skeletal muscle by >100-fold while markedly reducing expression heterogeneity [110,111]. Co-delivery of plasmid constructs enables concurrent gene upregulation and silencing, providing a combinatorial strategy to counteract muscle atrophy or injury [112,113,114]. The technique further permits real-time observation of subcellular protein dynamics in vivo when integrated with fluorescently labeled probes [115]. Notably, tissue damage may occur due to surgical intervention or high-field electrical exposure [116]. Furthermore, the enhancement of transfection efficiency through electroporation relies on the precise adjustment of electrical stimulation parameters specific to different skeletal muscles [111]. Additionally, studies have confirmed that electroporation subsequently activates immune pathways within the skeletal muscle [117]. This alteration in the immune status of the targeted tissue may influence the immune response between the vector and the host organism, thereby reducing transfection efficiency.

Another important physical delivery method in skeletal muscle delivery is ultrasound-mediated targeted delivery (UMTD). UMTD utilizes the energy of ultrasound waves in combination with contrast agents (such as microbubbles) to achieve the precise delivery and controlled release of therapeutic materials. Ultrasound waves induce oscillation or destruction of the microbubbles, generating mechanical forces that increase vascular permeability, promote drug release, and enhance tissue penetration [118]. Xi Xiang et al. leveraged UMTD to achieve spatiotemporally controlled delivery of IGF-1 and TGF-β to injured rat tendons, potentiating tendon regeneration and significantly attenuating fibrotic scarring [119]. Ultrasound-guided targeted delivery of PMO-containing microbubbles via intravenous administration significantly enhances skeletal muscle transduction efficiency, achieving substantial dystrophin restoration in *mdx* mice that is markedly superior to IV-injected PMO alone [120]. Compared to electroporation, UMTD exhibits reduced invasiveness to surrounding tissues while demonstrating comparatively lower transduction efficiency, requiring further optimization.

Beyond direct delivery of definitive-purpose DNA and molecular therapeutics to target organs, physically enhanced delivery systems synergize with AAV vectors or non-viral vectors to substantially potentiate transduction efficiency across diverse carrier platforms within tissues of interest. By integrating AAV-delivered large-fragment homologous donor constructs with electroporated Cas9/sgRNA ribonucleoproteins (RNPs), Sean Chen et al. developed the CRISPR-READI platform. This system achieved precise insertion of a 3.3-kb gene expression cassette into endogenous loci in mice, establishing a promising strategy for targeted large-fragment genomic integration [121]. Ultrasound-mediated delivery via liposomes and exosomes shows considerable potential, wherein ultrasonic cavitation enhances exosome enrichment in skeletal muscle and facilitates controlled drug release from liposomes. In skeletal muscle therapeutics, ultrasound stimulation selectively elevates circulating levels of muscle-derived exosomes whose anti-inflammatory miRNAs reshape the regenerative microenvironment [122]. Membrane-fusogenic hybrid exosomes (MFHE) combine the high drug-loading capacity of liposomes with the innate targeting of exosomes [123], while UMTD enhances delivery precision [124]. These integrated approaches establish novel non-invasive strategies for treating muscular injuries and dystrophies.

### 5.5. Antibody–Oligonucleotide Conjugate

Antibody-oligonucleotide conjugates (AOCs) are engineered by covalently tethering skeletal-muscle-targeting antibodies to therapeutic nucleic acids (e.g., siRNA, ASO), enabling receptor-mediated endocytosis via antigen binding on myocyte surfaces. This strategy amplifies the delivery efficiency of oligonucleotides to skeletal muscles [125]. Tsukasa Sugo et al. demonstrated that anti-CD71 antibody–oligonucleotide conjugates (AOCs) achieve potent gene silencing in cardiac and skeletal muscles. In murine peripheral artery disease models, myostatin-targeted AOCs induced selective silencing and elicited gastrocnemius hypertrophy, restoring exercise performance and validating the therapeutic potential of AOCs for muscular disorders [126].

## 6. Clinical Application

### 6.1. Gene Therapy Strategies

**Figure 5:**
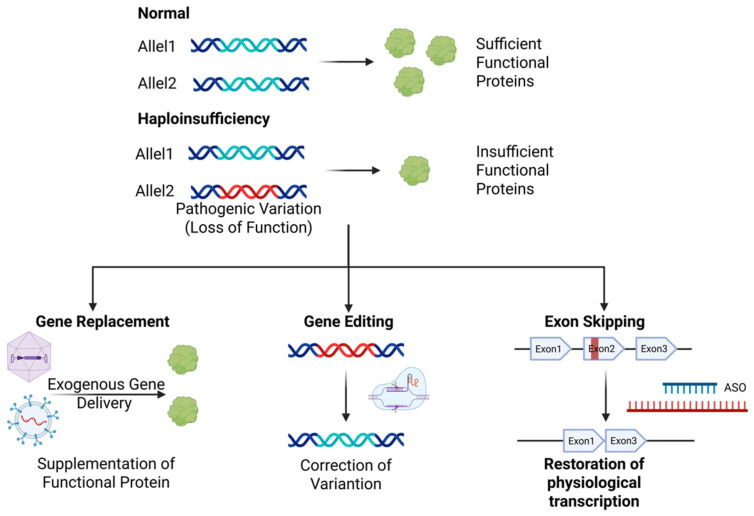
Therapeutic strategies for genetic therapy in hereditary myopathies. This schematic delineates core gene therapy approaches to address pathogenic mutations causing functional protein deficiency. Wild-type state: Biallelic expression yields sufficient functional protein to maintain cellular homeostasis. Loss-of-function (LOF) variation: Pathogenic variants (e.g., nonsense, frameshift) cause haploinsufficiency, resulting in insufficient functional protein production. Gene replacement therapy: Delivery of exogenous functional genes (e.g., via AAV or non-viral vectors) to restore physiological protein levels and compensate for endogenous deficiency. Gene editing; Precise correction of variations using CRISPR-Cas9 or base editors to reconstitute native gene function and enable endogenous production of full-length functional proteins. Exon skipping: Antisense oligonucleotides (ASOs) mediate the exclusion of variation-harboring exons during pre-mRNA splicing, restoring an open reading frame to generate truncated yet functional proteins.

In inherited myopathies, disease-causing genes exert their pathological effects through two primary mechanisms, either by producing insufficient wild-type protein for normal function or by expressing a harmful variant protein. Consequently, the core objectives of gene therapy strategies are to restore normal protein expression or reduce abnormal protein expression [127]. Therefore, the choice of gene therapy strategy depends fundamentally on the disease’s genetic mechanism and molecular pathology. Currently, three major therapeutic strategies have emerged, each possessing distinct mechanisms and specific applicability(Figure 5).

Gene replacement therapy compensates for defective gene function via functional gene supplementation, representing an optimal approach for monogenic recessive disorders, specifically for loss-of-function mutations. This strategy employs vectors to deliver normal gene copies to muscle cells, enabling sustained therapeutic protein expression. Classical gene replacement therapy predominantly employs AAV as the delivery vector. Zolgensma^®^ delivers the *SMN1* gene sequence via AAV9 to compensate for deficiency caused by *SMN1* variations [128]. Consistent with prior discussions of expression cassette engineering, proteins restricted by AAV packaging size constraints deliver truncated variants or employ intein-mediated full-length expression systems, as exemplified by Elevidys^®^ using AAVrh74 to deliver microdystrophin for DMD therapy [21]. Furthermore, current gene therapies delivering microutrophin (a dystrophin surrogate) demonstrate promising efficacy in DMD animal models with diminished immunogenicity [129]. Emerging non-viral vector-based gene replacement strategies are advancing rapidly, exemplified by the Shenzhen Bay Laboratory-led Phase IIT utilizing extracellular vesicles for full-length dystrophin mRNA delivery, which has secured regulatory clearance and entered preparatory implementation stages.

Gene editing therapies employ molecular tools to directly repair mutant loci in patient genomes, demonstrating particular utility for autosomal dominant or point-mutation-induced myopathies. Current widely utilized gene editing systems range from conventional CRISPR/Cas9 platforms and base editors (CBEs, ABEs) to nascent innovations, including prime editing (PE) technology [130]. Given the extensive spectrum and broad genomic distribution of genetic variations in hereditary myopathies, targeted correction of individual mutations demonstrates limited clinical translatability. Consequently, state-of-the-art gene editing therapeutics primarily achieve therapeutic outcomes through post-editing modifications, including exon skipping, exon insertion, frameshift restoration, and expression modulation, to functionally rescue pathological phenotypes. GEN6050X (ClinicalTrials.gov ID: NCT06392724), the world’s first DMD base-editing drug, utilizes cytidine base editing (CBE) to ablategenomic splice acceptor sites, thereby orchestrating exon 50 skipping in the *DMD* gene. This precision intervention restores wild-type dystrophin protein expression. Furthermore, therapeutic ABE and PE have established proof of concept in animal models for simultaneous *DMD* exon skipping and frameshift restoration [89,131]. Leveraging the single-nucleotide precision of base editors, high-frequency pathogenic variants in the *DMD* gene have been therapeutically corrected [132,133]. Utilizing gene editing to insert or replace mutated exons with intact ones is also a potential therapeutic strategy for hereditary myopathies [134]. Notably, an alternative therapeutic strategy in hereditary myopathies under development employs nuclease-deactivated Cas9 (dCas9) complexed with sgRNA to target the transcriptional start site of the wild-type allele. By tethering transcriptional activators (e.g., VPR tripartite effector), this approach achieves epigenetic amplification of endogenous protein expression, thereby restoring functional protein levels [135].

Exon skipping therapy, a variation-specific intervention for *DMD*, induces the modulation of pre-mRNA splicing to bypass deleterious variations, thereby enabling the restoration of partial dystrophin function. This approach selectively targets amenable variation subtypes to achieve internally truncated yet functional protein isoforms [136]. Antisense oligonucleotides (AONs), single-stranded DNA/RNA molecules optimized to 18-21 nucleotides, function as exon skipping therapeutic agents through Watson–Crick hybridization to target mRNA. This sequence-specific binding enables the modulation of splicing patterns, transcript stability, and translational efficiency, establishing AONs as first-in-class molecular therapeutics for targeted exon exclusion in precision genomic medicine [137]. The FDA has approved AONs, such as Eteplirsen (phosphorodiamidate morpholino oligomer targeting exon 51 of the dystrophin gene) and Golodirsen (similarly engineered for exon 53), as first-in-class splice-modulating agents, authorized under accelerated approval for frame-restoring dystrophin rescue in DMD patients with mutation-defined exon skipping eligibility [138,139].

### 6.2. Clinical Implementation Scenarios

Based on the aforementioned therapeutic strategies, numerous gene therapy agents leveraging AAV or non-viral delivery platforms have been clinically approved, are currently in active clinical trials, or are advancing through preclinical pipelines (Table 1).

AAV-based gene therapeutics have been clinically implemented across multiple hereditary myopathies. Elevidys^®^, the first FDA-approved DMD gene therapy, although failing to demonstrate statistically significant improvement in the primary endpoint (NSAA score), achieved sustained microdystrophin expression over 16 weeks and clinically meaningful benefits in the secondary endpoint Time to Rise (TTR), indicative of therapeutic bioactivity for phenotype modification [140]. A meta-analysis demonstrates that Elevidys^®^ therapy confers clinically meaningful benefits in DMD patients across functional metrics, including North Star Ambulatory Assessment (NSAA), Timed Functional Tests (TFTs), especially Time to Stand (TTSTAND) and 4-Stair Climb (TTCLIMB). Enhanced efficacy is observed in the 4–5-year-old cohort, supporting early intervention for superior therapeutic outcomes [141]. Zolgensma^®^ demonstrates significant restoration of motor function in SMA patients, achieving unprecedented clinical outcomes in severe cases, enabling ventilator-free survival and establishing functional motor milestones absent in natural disease progression [142,143]. Beyond these, numerous AAV-based gene therapies are under active clinical investigation as promising therapeutic platforms. AAV vector-mediated gene therapy demonstrates considerable therapeutic potential for hereditary myopathies, such as DMD and XLMTM [144]. However, significant safety concerns are associated with this approach, primarily manifesting as severe adverse reactions, including immune responses, liver injury, and infusion-associated reactions (IARs) [145]. Of these, immune reactions are the most prevalent and challenging. High-dose systemic administration can trigger robust innate and adaptive immune responses, leading to IARs characterized by fever and inflammation, which may be fatal in severe cases [146,147,148]. These immune reactions are also implicated in mediating dose-dependent hepatotoxicity or liver failure [147]. Furthermore, such dose-dependent immune events may involve coagulation dysfunction and complement pathway activation-driven systemic inflammation [145]. Pre-existing AAV antibodies can exacerbate these immune reactions [149]. These adverse effects have resulted in confirmed fatal outcomes, including the death of a patient receiving CRISPR-AAV gene therapy in a DMD clinical trial, the unfortunate death of three children following AAV8 gene therapy in an XLMTM trial, and fatal events due to immune or toxicology mediated multi-organ (liver, kidney, heart, lung) failure observed during high-dose AAV therapy for neuromuscular disorders [146,147].

Distinct from AAV gene therapy, non-viral vector gene therapy strategies are continuing to emerge. Historically, AONs have shown the most established therapeutic efficacy for inherited myopathies among non-vital vector approaches. AONs are currently utilized across multiple disease therapies. Exemplified by Nusinersen for SMA therapy, this AON demonstrated statistically significant efficacy and a favorable safety profile in phase III clinical trials. It successfully enhanced motor milestone achievement and prolonged survival outcomes, establishing paradigmatic success in oligonucleotide therapeutic development [150]. Beyond SMA, a steady progression of novel AON therapeutics is advancing into clinical trials with regulatory clearance [151]. Current clinical trial data demonstrate that AON therapies for DMD achieve only minimal restoration of dystrophin protein expression, with no statistically significant functional improvements observed [152,153]. This limited clinical efficacy stands in contrast to preclinical models where even trace dystrophin recovery corrected disease phenotypes [154]. Substantial treatment heterogeneity is documented, where baseline motor function, age, and mutation genotype significantly predict therapeutic response trajectories [152,155]. Furthermore, observed nephrotoxicity and other potential off-target organ toxicities necessitate expanded safety datasets for comprehensive risk profiling. Non-viral vectors (exosomes/liposomes/polymers) currently show preferential applications in oncology and diabetes [156,157]. These platforms have validated clinical utility, evidenced by systemic LNP-mRNA therapy curing CPS1 deficiency through in vivo base editing [158]. A Shenzhen Bay Laboratory-led Phase IIT clinical trial is now exploring extracellular-vesicle-delivered full-length dystrophin mRNA, accelerating non-viral gene therapy development for hereditary myopathies with multidirectional delivery approaches.

### 6.3. Cardiac Tropism in Gene Therapy

Hereditary myopathies frequently compromise cardiac muscle, necessitating genotherapeutic vectors with co-optimized tropism for both skeletal myofibers and cardiomyocytes to achieve comprehensive therapeutic efficacy [159,160]. Despite native AAV9 tropism for both cardiac and skeletal muscle, its clinical utility is constricted by suboptimal transduction efficiency, profound hepatotoxicity, and pre-existing immunogenicity, limiting re-administration [161,162]. Through AAV capsid engineering, contemporary AAV vectors demonstrate substantially enhanced tropism, simultaneously achieving selective transduction of both cardiac tissue and skeletal musculature. Hichem Tasfaout et al. achieved dystrophin restoration in critical DMD pathology sites, including the skeletal muscle, diaphragm, and myocardium, through triple AAVMYO1 delivery with split intein splicing technology [163]. Compared to wild-type AAV9, the engineered AAV-MYO2/MYO3 platforms exhibit markedly attenuated hepatic off-targeting with concurrent enhancement in dual-tissue tropism, achieving superior cardiomyocyte transduction and skeletal muscle selectivity in mdx mice [51]. Beyond the AAVMYO series, engineered capsids (e.g., MyoAAV series, LICA-AAV) demonstrate concomitant cardioskeletal tropism enhancement with attenuated hepatic off-targeting compared to AAV9 [44,50]. Current non-viral platforms remain skewed toward skeletal-muscle-selective delivery, whereas dual-tissue targeting vectors for simultaneous myocardial and skeletal transduction remain unexplored. Optimizing bimuscular specificity in genetic myopathy therapies critically balances efficacy–toxicity ratios, particularly in DMD, where pan-musculature therapeutic coverage confers more comprehensive protection [26].

### 6.4. Comparison of AAV and Non-Viral Vectors

In the field of skeletal muscle gene therapy, AAV vectors remain the predominant delivery platform, serving as the primary choice for therapeutic gene transfer. Among natural AAV serotypes, AAV1, AAV6, AAV7, AAV8, and AAV9 exhibit intrinsic myotropic properties, with AAV9 demonstrating unparalleled transduction efficiency in skeletal and cardiac muscle tissues [164]. Following nuclear entry into myocytes, the single-stranded DNA genome of AAV vectors predominantly persists as episomes without integrating into host chromosomes. This non-integrating property enables long-term stable transgene expression lasting from months to years in long-lived post-mitotic myofibers after a single administration [165]. Engineered AAV9 (eAAV9) vectors targeting integrin αVβ6 or insulin receptors represent a mature modification strategy, demonstrating significantly enhanced myotropic transduction efficiency in skeletal muscle compared to wild-type AAV9, primarily through ligand-receptor-mediated endocytosis and tissue-specific tropism refinement [44,166]. Despite their therapeutic promise, current AAV vectors face several inherent limitations. Systemic administration of AAV vectors can cause off-target organ infection and toxicity, representing a major constraint of their clinical utility. And, a significant proportion of the population harbors pre-existing neutralizing antibodies (NAbs) against common AAV serotypes, which can ablate initial transduction efficacy upon first administration. Additionally, both AAV capsid proteins and foreign transgene products may elicit robust cellular and humoral immune responses in the host, potentially eliminating transduced cells and precluding redosing due to immunological memory [167,168]. The limited packaging capacity stated before restricts large protein expression application. The AAV manufacturing process, which involves multiple intricate steps, including cell culture, transfection, and viral purification, characterized by its complexity, time-consuming nature, and exorbitant costs, significantly restricts its widespread clinical adoption [169].

Compared to AAV, non-viral vectors are emerging as novel gene delivery systems. Non-viral vectors are primarily composed of synthetic materials or endogenous substances, exhibiting superior biocompatibility and the capacity to effectively evade immune recognition and clearance, thereby posing minimal risk of immune rejection [122,170] (Table 2). Non-viral vectors exhibit significantly larger payload capacity than AAV, enabling the delivery of larger therapeutic proteins, such as full-length dystrophin. Non-viral vectors exhibit a modular design architecture, enabling straightforward integration of diverse modifications to confer multiple functionalities. Streamlined production processes further constitute a key advantage of non-viral vectors over AAV. However, non-viral vectors face multiple biological barriers in vivo, including the extracellular matrix, nuclease degradation, cellular endocytosis, and endosomal escape, resulting in significantly lower gene delivery efficiency compared to AAV. The delivered nucleic acids typically exist in a non-integrated form within cells and are prone to degradation, leading to transient transgene expression that necessitates frequent administration to maintain therapeutic efficacy. Furthermore, systemic application of non-viral vectors fails to achieve homogeneous transduction across systemic muscles, particularly in cardiac and diaphragmatic tissues, unlike engineered AAV variants.

## 7. Future Perspectives

Despite its current limitations, gene therapy as a highly promising therapeutic approach has spurred the development of numerous refined strategies to address these challenges of its vectors.

Future innovation of AAV vectors centers on engineered capsid modifications. AI-driven design of novel serotypes significantly enhances skeletal muscle targeting while evading immune clearance by pre-existing neutralizing antibodies. Concurrent development of tissue-specific promoters, such as the muscle-restricted MHCK7 promoter, strictly confines transgene expression to minimize off-target organ damage. To address immunogenicity challenges, rational modifications to capsid surface structures reduce innate immune recognition, complemented by adjunctive therapies like plasmapheresis for antibody clearance, and complement inhibitors for acute toxicity control. For highly immunogenic transgenes (e.g., full-length dystrophin), truncated variants or species-homologous sequences enhance immune tolerance without compromising functionality. AI further empowers the entire development pipeline, predicting capsid–receptor interactions, optimizing off-target effects, and simulating production parameters, accelerating clinical translation.

Non-viral vector advancements rely on material innovation and functional integration. Modular design enables the incorporation of multiple elements; targeting peptides enhance muscle-specific accumulation, stealth coatings evade host clearance, stimuli-responsive components enable microenvironment-triggered release, and nuclear localization signals guide efficient nuclear entry. Virus-like particles (VLPs) delivering CRISPR ribonucleoproteins achieve >80% editing efficiency while avoiding genomic integration risks. Hybrid strategies, such as AAV–exosome systems, synergize exosomal immunomodulatory properties with AAV’s high transduction efficiency, markedly improving targeting precision and reducing immune clearance.

Industrial-scale progress necessitates standardized production and cost control. Microfluidic continuous-flow processes minimize batch variability in viral vectors, while thermostable lipid nanoparticles (LNPs) overcome cold-chain limitations, ultimately reducing costs to ~USD 10,000 per dose. Clinical translation requires dynamic dose–immunity–efficacy balancing models, long-term safety monitoring networks, and biomarker-based patient stratification to improve the therapeutic effect of gene therapy drugs. Payment innovations and global regulatory harmonization will enable the transition from single-treatment interventions to lifelong cures, redefining hereditary myopathies’ management.

Vector technologies are evolving from mere delivery tools to programmable therapeutic systems. The convergence of viral and non-viral platforms, powered by AI-driven high-throughput design, standardized manufacturing, and precision clinical strategies, will realize the vision of “single-administration, lifelong cure” for muscular disorders.

## Figures and Tables

**Table 1:** FDA-approved gene therapies for hereditary myopathies.

Therapy Type	Drug Name	Target Disease	Mechanism	Status
Gene Replacement	Zolgensma^®^ (onasemnogene abeparvovec)	Spinal muscular atrophy (SMA)	AAV9-delivered SMN1 gene	Full approval (2019)
	Elevidys^®^ (delandistrogene moxeparvovec)	Duchenne muscular dystrophy (DMD)	AAVrh74-delivered microdystrophin	Traditional approval (2024)
Exon Skipping	Eteplirsen	DMD (exon 51 amenable)	PMO-ASO skipping exon 51	Accelerated approval (2016)
	Golodirsen	DMD (exon 53 amenable)	PMO-ASO skipping exon 53	Accelerated approval (2019)
	Viltolarsen	DMD (exon 53 amenable)	PMO-ASO skipping exon 53	Accelerated approval (2020)
	Casimersen	DMD (exon 45 amenable)	PMO-ASO skipping exon 45	Accelerated approval (2021)

**Table 2:** Comparative analysis of AAV vs. non-viral vectors for skeletal muscle gene therapy.

	AAV Vector	Non-Viral Vector
Transduction efficiency	High	Low
Duration of transgene expression	Sustained expression	Transient expression
Immunogenicity	Prone to eliciting	Less likely to induce
Scalability of manufacturing	Higher manufacturing complexity	Streamlined production processes
Development complexity	Poses significant development challenges	Amenable to rapid development
